# An activity analysis of Dutch hospital-based physician assistants and nurse practitioners

**DOI:** 10.1186/s12960-019-0423-z

**Published:** 2019-10-29

**Authors:** G. T. W. J. van den Brink, A. J. Kouwen, R. S. Hooker, H. Vermeulen, M. G. H. Laurant

**Affiliations:** 1 Radboud Institute for Health Sciences, IQ healthcare, Radboud University Medical Center, Nijmegen, The Netherlands; 2 Department of Master Programs, HAN University of Applied Sciences, PO box 6960, 6503 GL Nijmegen, The Netherlands; 3 Radboud University Medical Center, PVI, Nijmegen, The Netherlands; 4 Health Policy Analyst, Ridgefield, WA United States of America; 5 HAN University of Applied Sciences, Institute of Nursing Studies, Nijmegen, The Netherlands

**Keywords:** Task transfer, Task shifting, Skill mix, Substitution, Delegation, Professional role, Hospital administration

## Abstract

**Background:**

The physician assistant (PA) and the nurse practitioner (NP) were introduced into The Netherlands in 2001 and 1997 respectively. By the second decade, national policies had accelerated the acceptance and development of these professions. Since 2015, the PA and NP have full practice authority as independent health professionals. The aim of this research was to gain a better understanding of the tasks and responsibilities that are being shifted from Medical Doctors (MD) to PAs and NPs in hospitals. More specifically in what context and visibility are these tasks undertaken by hospital-based PAs and NPs in patient care. This will enable them to communicate their worth to the hospital management.

**Study design:**

A descriptive, non-experimental research method design was used to collect and analyze both quantitative and qualitative data about the type of tasks performed by a PA or NP. Fifteen medical departments across four hospitals participated.

**Methods:**

The patient scheduling system and hospital information system were probed to identify and characterize a wide variety of clinical tasks. The array of tasks was further verified by 108 interviews. All tasks were divided into direct and indirect patient care. Once the tasks were cataloged, then MDs and hospital managers graded the PA- or NP-performed tasks and assessed their contributions to the hospital management system.

**Findings:**

In total, 2883 tasks were assessed. Overall, PAs and NPs performed a wide variety of clinical and administrative tasks, which differed across hospitals and medical specialties. Data from interviews and the hospital management systems revealed that over a third of the tasks were not properly registered or attributed to the PA or NP. After correction, it was found that the NP and PA spent more than two thirds of their working time on direct patient care.

**Conclusions:**

NPs and PAs performed a wide variety of clinical tasks, and the consistency of these tasks differed per medical specialty. Despite the fact that a large part of the tasks was not visible due to incorrect administration, the interviews with MDs and managers revealed that the use of an NP or PA was considered to have an added value at the quality of care as well to the production for hospital-based medical care in The Netherlands.

## Background

A growing number of countries have expanded their medical services by incorporating the nurse practitioner (NP) or physician assistant (PA) [3, 12]. For the most part, the reason is the increasing demand of healthcare due to a rising number of chronically ill patients, comorbidity, and an aging population [27, 30]. Added to this social burden are growing costs of care, rising patient expectations, emerging technologies, and treatment opportunities. In turn, the demand of healthcare places pressure on governments and medical institutions to develop more effective and high-quality delivery systems [15].

The incorporation of PAs and NPs on medical teams is evolving yet at the same time their inclusion seems to be a good fit. Both appear to be well suited to assume medical tasks that, at one time, were exclusively performed by physicians [13, 16, 36]. Furthermore, the growing presence of PAs and NPs in North America and Europe suggests these are valued human resources readily available to accept the challenges of rising demand for medical services [4, 9, 21]. However, to date, an inventory of tasks and responsibilities of NPs or PAs in hospital roles has only been recently documented [31]. How they perform in direct patient care remains an area of interest to health workforce researchers and health care managers [9, 33]. For the development of new professions, it is important that their contribution be visible [12], Allen 2015, [9]. After all, descriptive and result-oriented work quantifications are necessary to communicate their worth to the patient care [3].

Healthcare administrative systems can provide an important perspective about the tasks and responsibilities of their employees and are therefore more frequently used in health care research [23].

This paper reports on tasks and responsibilities of Dutch PAs and NPs employed by hospitals. Since their introduction in The Netherlands, the number of PAs has grown from 347 in 2012 to 1231 in 2019 and the number of NPs increased from 1307 to 3672 in 2019 [5, 25, 32]. As of 2019, there were 70 000 registered physicians [5]. Since the introduction of the NP and PA, a series of studies have assessed the role, responsibility, and value to Dutch society [8]. The Dutch Healthcare Authority in 2015 introduced a policy warranting that hospitals be reimbursed for the activities performed by a PA or an NP. This policy requires an accurate report of tasks and responsibilities of NPs and PAs. Nonetheless, it is unclear whether healthcare administrative systems are indeed accurate in showing tasks and responsibilities in medical care. Because their effect on medical services in hospitals has been only been marginally described, we undertook an inventory of the tasks performed by NPs and PAs in four Dutch hospitals. Our aim was to:

- Describe tasks performed by PAs and NPs in hospitals,
- Categorize patient and non-patient-related tasks,
- Describe how the supervision and collaboration was organized and what the contributed value of the PA and NP was, and
- Assess the reliability of hospital administrative systems to capture the activity of PAs and NPs.

The intent of this study was to gain insight into the tasks that have been shifted from MDs to PAs and NPs. More importantly, how this task shifting is being valued and how visible the contribution is in the hospital-based management information systems.

## Theoretical framework

Based on the literature and discussions with health workforce researchers, the concept of medical tasks being shifted from doctor to PAs or NPs was cataloged into four categories: substitution of tasks, delegation of tasks, additional tasks, and other tasks [21]. “Substitution of tasks” is defined as a structural transfer of assignments from physicians to any health professional (Table 1). The one assuming the task is responsible for the task. Which medical task a NP or PA performs is the result of consultation with the MDs (the doctor or the medical manager). In older literature, the term “delegation” was used to describe the transfer of physician-substituted roles and procedures and viewed as a labor economic term [28]. “Delegation” in this sense was that the health professional performs the task under supervision; the physician gives specific directions how to perform the task and the physician remains responsible for the task [6, 28].

**Table 1:** Types of tasks undertaken by PAs and NPs in four Dutch hospitals

1	*Substitution (transfer of tasks)* is aimed at a structural transfer of tasks. This means tasks are carried out autonomously, the tasks are part of standard scheduling, and the NP or PA is considered to be fully responsible for the “transferred” task.
2	*Delegation* is the incidental transfer of tasks. It involves entrusting certain tasks to the NP or PA. In this respect, the temporary nature as well as the direct involvement of the physician (MD) is crucial, i.e., the task is not routinely planned and there is the possibility of direct supervision and intervention by the MD. The task is performed on behalf of the MD.
3	*Additional tasks* are an extension of the tasks of existing professionals. In this case, a distinction is made between “patient-related” and “non-patient-related” to point out the difference between, for example, psycho-social care and administrative/logistic tasks.

## Methods

### Study design

A combination of quantitative and qualitative research methods was used to gather information about the tasks shifted from a medical doctor to an NP or PA. This included financial administrative system data, roster information, outpatient appointment schedules, and a questionnaire with open and closed questions for NPs and PAs, along with semi-structured interviews involving MDs, managers, PAs, and NPs.

### Setting

Dutch hospitals that employed PAs and NPs were invited to participate in this research. Four hospitals met the following criteria:

1. Access to the financial system from which data could be extracted in such a manner that the activity was discernable per patient (diagnosis-treatment combination) and the provider could be identified;
2. There were no legal, moral, or technical obstacles that inhibited sharing the data with researchers.
3. Provide care to patients with both acute and chronic illnesses and have a variety of medical specialties.

Five hospitals were invited to participate (purposeful sample) and four enrolled in the study: three general hospitals and one university hospital.

### Data collection and data analysis

**Fig. 1:**
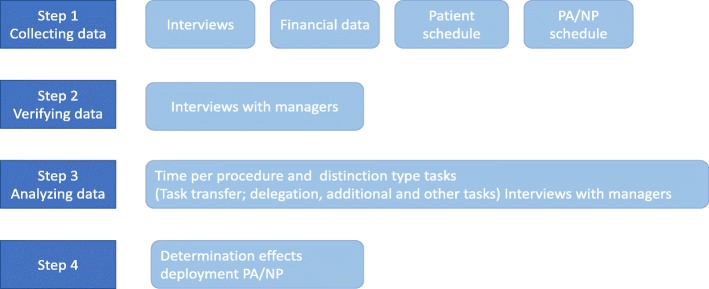
Data collection and analysis

We collected the information on all the tasks executed by an NP or PA, categorized the tasks, and analyzed the data. At the same time, we documented the time needed to perform the tasks and compared times with a physician normally executing the tasks, along with the time needed for physician supervision. Data collection and analysis followed a four-step approach (see Fig. 1).

#### Step 1

Information was collected about the productivity of PAs and NPs from November 2015 to June 2016. Medical specialty data was obtained from the appointment schedules for outpatients and financial information from the hospital administrative systems. Concurrently, interviews with employed PAs and NPs were undertaken regarding their role, tasks, and productivity. Together with the questionnaires, the collected data resulted in a list of procedures and tasks involving patients treated by the NP or PA. Next, the recorded procedures and duration of the time with the patient were used to quantify the encounter. Any mismatch between the data from the administrative system and the outpatient schedule was reconciled by contacting the supervising medical specialist and/or financial data administrators at the hospital. In the catalog of procedures and tasks, we included “additional tasks.” Additional tasks were those that were new as well as other tasks that could not be categorized from the hospital management system.

#### Step 2

**Fig. 2:**
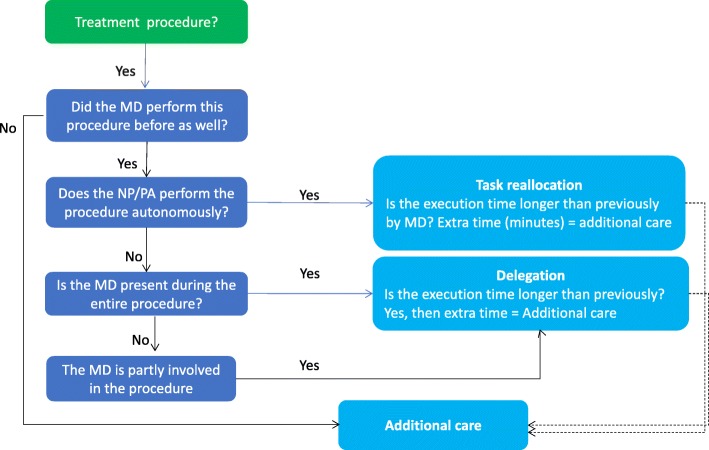
Task analysis flowchart identifies the data collection process

Trained researchers interviewed 35 clinic or department managers in the four hospitals. Collectively, the managers were responsible for the planning and control of daily activity and finances within the hospital or medical specialty departments. The interviews centered on the productivity of the hospital-based PA or NP. The managers and MD were asked to rate the overall contribution of the PA or NP in terms of quality of patient care and production on a visual analog scale from 1 to 10. Three researchers then independently analyzed the results, by following the algorithm from Fig. 2 and reconciled any differences into one list. Next, we inventoried how many minutes the physician provided supervision for every procedure the NP or PA performed. Supervision was defined as instructing, collaborating, or overseeing the procedure. For validation purposes, the indicated time for executing a procedure with the patient was verified with the schedule of outpatient appointments; also assigned was the time the procedure started and when completed. In this way, we classified the degree of autonomy from the supervising medical doctor, triangulated with the patient’s record (Fig. 1). To distinguish the tasks, descriptive statistics were incorporated into:

- Type of task transfer,
- Number of tasks and activities,
- Duration of the execution of the task and needed supervision.

#### Step 3

The collected data were put into an Excel database. Included were detailed information about the tasks and the procedures performed by the PA or NP, along with any distinction between the recorded number of procedures in the outpatient schedule data and the performed procedures as reported by NP or PA. The data of the inventoried tasks were divided into three categories: substitution of tasks, delegated tasks, and additional tasks. These three categories were assigned a degree of independent performance based on what the PA or NP said and corroborated by the MD. The time to perform the task was stated in minutes using the electronic system (see Fig. 2). All other activities not recorded in the hospital electronic information system, but mentioned in the interviews, were classified as overhead or “other tasks.”

#### Step 4

The collaborating MD of each PA or NP assigned to the department was also asked broadly about the technical nature of the procedure. When inconsistencies emerged, additional information about the issue or task was reconciled by discussing the topic with the PA or NP, MD, and someone within the administrative system. In the interviews, we also asked what contribution the NP or PA added was in terms of quality of care and any contribution or value added to the production and efficiency of the service.

## Results

The four included hospitals, from different regions in The Netherlands (south, east, west, and center of the country), differ from production, turnover, and number of staff. In this way, a representative selection has been made. We used the data from 75 NPs/PAs (that was 57% of the total population of PAs and NPs employed by the four hospitals at the time of the study). We interviewed 38 MDs and 20 managers. Also of the 75 NPs/PAs, we selected 32 NPs and 21 PAs for interviews, per participating department only 1 NP and 1 PA. Based on a comparison of the characteristics of the interviewees such as average age, experience as NP or PA, and the total work experience in health care with the characteristics of a national inventory among alumni [25, 32], we included a representative sample of NPs and PAs. The mean working hours per year for these NPs and PAs were, respectively, 1381 (SD 238) and 1502 h (SD 272) (Table 2). In total, 2883 h of the included PA/NP time was assessed over 8 months.

**Table 2:** Number of hours spent on tasks (based on 8 months). Financial administration records combined from all four institutions

	NP (*N* = 32)		PA (*N* = 21)	
	Hours	%	Hours	%
Task substitution	309	22	465	31
Task delegation	52	4	34	2
Additional tasks	254	18	128	9
Other tasks	766	55	875	58
Total	1 381	100	1 502	100

The number of hours spent on tasks was parsed into the four task categories. Task substitution was 22–31%, task delegation was 2–4%, and “additional tasks” was 9–18%. According to the four hospital-based administrative systems that documented their activity, NPs and PAs spent more than half their time on “other tasks” (55–58%).

### Task transfer

**Fig. 3:**
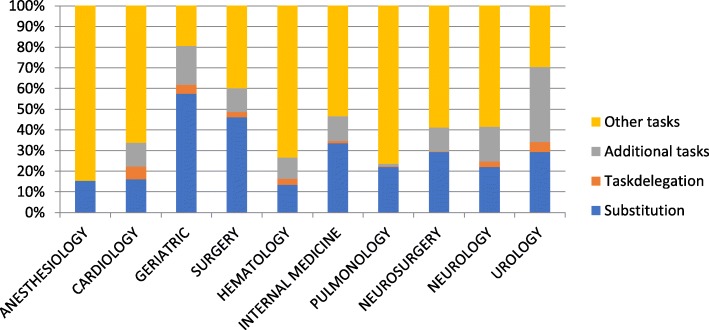
Differences between medical specialty departments

When the tasks were delineated into departments or medical specialties, there were wide variations in the categorization of tasks among the different medical specialties where the PA or NP was active (Fig. 3). The greatest task substitution was in geriatrics (58%) and the least in hematology (13%). Across specialties, the maximum part of the activities of an NP/PA was classified in the broad category of “other tasks.”

When profiles derived from hospital administrative systems were adjusted with the outpatient schedule and the data from the interviews correlated, what emerged was that “other tasks” were mainly those involving an aspect of direct patient care such as prescribing or arranging some patient accommodation. These tasks were moved into the category of “task substitution” because these activities were undertaken by physicians prior to the incorporation of PA or NP (Table 3). When patient-based (i.e., substitution, delegation, and additional tasks) and non-patient-based tasks were further parsed, on average, 46% of “other tasks” were related to direct patient care (Table 4). However, these were tasks not visible from the administrative record of physician activity. When these tasks were re-categorized to task substitution, an NP spent, in total, 73% on patient-based care and a PA 71%.

**Table 3:** Hours spent on tasks (based on 8 months) after re-categorization of other tasks to task substitution based on interviews

	NP (*N* = 32)		PA (*N* = 21)	
	Hours	%	Hours	%
Task substitution	708	51	906	60
Task delegation	52	4	34	2
Additional tasks	253	18	129	9
Other tasks	368	27	433	29
Total	1 381	100	1 502	100

**Table 4:** Other tasks

	NP	PA
	Hours	Hours
Administrative	61	91
Research	44	48
Expertise enhancement	40	38
Organizational tasks	36	38
Education (teaching)	40	34
Consultation between medical specialist and PA/NP	24	48
Remainder of the group	89	111
Total hours	368	433

The additional “other tasks” or administration tasks mentioned in the interviews were further delineated into:

- Requests for laboratory tests,
- Arranging appointments,
- Consultation (not about individual patients), planning, discharge, etc.

“Other tasks” also included clinical research, education/professional development, organizational tasks, education/teaching, and intercollegiate consultation (Table 4).

### Supervision and collaboration

The analysis of tasks also revealed that the presence of the physician overseeing the PA or NP was only reported a third of the time. For the NP, it was 64% of the cases and for the PA in 68% of the cases that they executed the task or procedure autonomously (without supervision or consultation). If there was a consultation with the MD, the average time was 6 min (SD 1.91). In regard to consultant availability, 36% of the NPs, 64% of the PAs, and 40% of the MDs concluded that “consultation between a PA or NP with the MD should always be made available” when requested.

The interviews revealed that an NP or PA, on average, was scheduled for a longer period of time for a patient consult than the physician: an NP 15 min (SD 2.53) longer and a PA 7.5 min (SD 2.57) longer. The PAs and NPs claimed that they provided the patient more information because the patients asked more questions than when the MD was the proceduralist. The time spent on providing additional information to patients was categorized as “additional tasks.” Some NP/PAs respond in the interviews that they needed more time per consult because they had no assistance from a medical assistant. At the same time the managers and MDs offered that the deployment of the NP or PA enhanced the quality of patient care and improved the production and efficiency of the medical service.

## Discussion

This description, assessment, and quantification of tasks of hospital-based PAs and NPs was based on documented procedures and interviews about the procedures. Of those procedures assessed in this 8-month time frame, NPs performed 26% of all the medical tasks recorded in a systematic way, and PAs 33% (task substitution and delegation together, see Table 2). The interviews and validation process (triangulation as described in step 4 of the data analysis) revealed that there was a relatively low registration or documentation of clinical tasks prior to completion of this study. This omission was largely attributed to hospital policies or procedures that were inconsistent and not standardized in how they were recorded. In fact, once the data was reconciled, the PA performed 62% and the NP 55% of their working time on clinical tasks that previously had been performed exclusively by physicians (i.e., task substitution and delegation combined, see Table 3).

Another finding was the division of labor between PAs and NPs. In this study, the results show some minor differences between the PAs and NPs. The PA appeared to be performing clinical tasks more independently than NPs; however, these differences were not statistically analyzed, but their similarity and interchangeability has been noted by other observers [14]. Furthermore, the medical and administrative staff regarded both professions equally and did not see much difference. This was because, in part, both spent a large part of their working time on direct patient care. Time-motion studies are needed to better quantify how PAs and NPs function in hospital settings [2, 26].

What PAs and NPs do, how well they do it, and what impact this has on patient-centered results are a needed piece of health service research [11, 19, 20, 24, 31]. One finding in this study revealed that a great number of tasks performed by a PA and NP in Dutch hospitals were not visible to administrators due to lack of documentation or registration. The reasons were:

- The administrative systems in the hospitals were not consistently prepared for PAs or NPs that performed independently tasks or procedures.
- PAs and NPs were not always able or willing to fill in the information into the hospital informatics system.
- Sometimes a medical specialty had a policy that did not permit a PA or an NP to document the tasks or procedures.
- PAs and NPs performed a great deal of overhead tasks that do not exist or did not have a category in the administrative system.

These tasks can be described as *patient-centered clinical management.* Such tasks appear to contribute to the continuity of care (facilitating patient flow, an easier access for nurses to the medical team, and more information for the patient and their next of kin). The additional set of medical providers seem to connect healthcare professionals around patients and their families and are perceived by the staff as a safety net for everything that needs to be aligned and coordinated. These findings are quite similar to the findings of Drennan et al. who researched the role of physician associates in secondary care in the United Kingdom [9]. To paraphrase, the NPs and PAs improve hospital functioning with their low visibility of tasks, but are missed when absent [12], (Allen 2015).

The outcome of care by a PA or NP, in terms of quality of care, as well as any contribution or value added to the production and efficiency of the care, is regarded at the same level as a MD based on a large number of observations that tend to transcend time, country, and type of patient [2, 9, 16, 22, 26, 31]. The shifting of clinical tasks from physicians to PAs or NPs was one of the main goals for the introduction of these professions and remains an important component of their visibility and development [10, 28, 29]. Where there is low visibility of the NP and PA contribution to the medical care, there cannot be an objective recognition [26]. Without recognition, there is the danger that the development of a relatively young profession will be undermined [12].

## Methodological considerations

The strength of this study lies in its novel method of understanding the concept of shifting clinical tasks in hospital settings. The use of an administrative approach to obtain a broad overview of task activity was needed as a first foray into this unknown area of medical labor research. Administrative data is a starting point for investigation of role activity because it can serve as a contrast to self-reported data in surveys and interviews—which is retrospective and assumed to be vulnerable for recollection bias. However, this assumption has not been well tested—especially as it applies to PAs and NPs. A flexibility of methods has been promoted in administrative research by Lazarfeld (1993) and continues today when public and government are involved in funding policy initiatives [7]. One aim is to not only interview different professionals and managers but also gather objective data from outpatient schedules and the financial registration needed for correlation and validation purposes. The files of these different hospital sources were integrated with data analyses and crosschecked during data collection. Discrepancies in the financial system capture of reimbursable procedures, outpatient schedules, and interviews were discussed with the managers and supervising medical specialists along the way. By using the results of the interviews and the data from the administrative system along with representative patient planning activities, the research team was able to objectify that the PA or NP may have been acting as contributors to a more efficient hospital service delivery. Through this triangulation and analyzing data as a whole, we reduced the chance of information and recall bias.

There are a number of limitations of this study. First is that the research was confined to four hospitals. Furthermore, the contributions of the PAs and NPs were measured by interviewing the professionals but at the same time revealing that the registration of tasks in the financial system was not always properly documented. Patient satisfaction was only researched indirectly as the study did not include patient impressions. However, we believe the stage is set with this study for a broader investigation that would include acceptance and satisfaction of patients by an array of providers undertaking various tasks.

## Conclusion

The World Health Organization (WHO) has identified “task shifting” or “task transfer” as the rational redistribution of tasks among health workforce teams [34]. Globally, the introduction of PAs and NPs, in terms of positioning and contribution, has resulted in a wide variety of roles including hospital employment. Our research revealed that PAs and NPs based in hospitals were taking on more clinical tasks than could be derived from the management system alone because the documentation of these tasks was inadequate or ineffective. At the same time, managers and MDs reported appreciating the contribution of their skills, availability to offset tasks, and providing a team-based approach to healthcare. Especially, the tasks that help the patient flow are very important but were not visible. The contribution of NPs and PAs in the direct patient care has become more visible which in turn leads to more reliable assessment of the activities as an important condition for the communication about their worth to the hospital and a further implementation of these professions in the Dutch healthcare system.

## Data Availability

Data files are available from the authors on reasonable request.

## Abbreviations

MD: Medical doctor
NP: Nurse practitioner
PA: Physician assistant
WHO: World Health Organization
